# The use of modafinil for the treatment of fatigue in multiple sclerosis: A systematic review and meta‐analysis of controlled clinical trials

**DOI:** 10.1002/brb3.3623

**Published:** 2024-07-10

**Authors:** Shamas Ghazanfar, Minaam Farooq, Shurjeel Uddin Qazi, Bipin Chaurasia, Ulrike Kaunzner

**Affiliations:** ^1^ Department of Medicine Dow University of Health Sciences Karachi Pakistan; ^2^ Department of Neurological Surgery King Edward Medical University Lahore Pakistan; ^3^ Department of Neurosurgery Neurosurgery Clinic Birgunj Nepal; ^4^ Department of Neurology Weill Cornell Medical College New York New York USA

**Keywords:** adverse events, fatigue, MFIS, modafinil, multiple sclerosis, quality of life

## Abstract

**Introduction:**

Multiple sclerosis (MS) is a debilitating neurological condition affecting nearly one million people across the United States. Among the most prominent symptoms of the condition are excessive fatigue and daytime sleepiness. Numerous clinical trials have investigated the efficacy of modafinil in addressing fatigue among these patients.

**Objective:**

The objective of the present study is to assess the safety and efficacy of modafinil for the treatment of fatigue in MS.

**Methodology:**

An electronic search of PUBMED, ScienceDirect, and Cochrane Central was conducted for articles published from inception to December 2023 using search terms such as “modafinil,” “fatigue,” and “MS.”

**Results:**

Seven studies were included in our analysis. Modafinil leads to a meaningful reduction in fatigue when compared with placebo, as measured by Modified Fatigue Impact Scale [mean difference (MD) = −4.42 [−8.01, −.84]; *I* ^2 ^= 45%; *p* = .02] and Epworth Sleepiness Scale [MD = −.87 [−1.64, −.10]; *I* ^2 ^= 0%; *p* = .03]. Modafinil also demonstrated a greater risk of precipitating adverse events (e.g., insomnia, gastrointestinal symptoms) when compared with placebo [RR = 1.30 [1.03, 1.66]; *I* ^2 ^= 0%; *p* = .03]. In quality‐of‐life assessments, modafinil was associated with overall improvement in well‐being [standardized mean difference = .18 [.01, .35]; *I* ^2 ^= 56%; *p* = .04].

**Conclusion:**

The data indicates that modafinil confers a therapeutic benefit when treating fatigue in patients with MS and improves overall quality of life; however, there is a risk of precipitating adverse events. Ultimately, higher quality of evidence may be required to better inform clinical management.

## Graphical abstract

The objective of the present study is to assess the safety and efficacy of modafinil for the treatment of fatigue in multiple sclerosis.

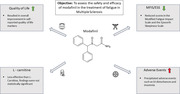

## 1 INTRODUCTION

Multiple sclerosis (MS) is a debilitating neurological condition that affects nearly one million people across the United States (Wallin et al., 2019). This autoimmune condition predominates in women who present with a variety of neurological symptoms including weakness, numbness, vision symptoms, bladder dysfunction, and severe generalized fatigue. The majority of patients present with a relapsing‐remitting disease course, which can advance into secondary progressive MS. A less common subtype is primary progressive MS, which occurs in about 15% of patients. The treatment for MS is primarily concerned with the use of immunomodulating and immunosuppressive medications, which help reduce inflammation and limit disease progression; however, these agents often come with a risk of adverse reactions, including possible infections (Ghasemi et al., 2017). More recent disease modifying treatments have shown great promise in the treatment of the disease, with some even striving toward the outcome known as no evidence of disease activity, which is based on a demonstration of low clinical measures or lack of disease symptoms, relapses, progression, and MRI activity (Newsome et al., 2023). Yet, despite the present advancements, the symptoms of fatigue and lethargy that are experienced by those with MS stand as a crucial obstacle in the way of substantial improvement in the quality of life (QoL) for sufferers (Braley & Chervin, 2010).

Modafinil, otherwise known as diphenylmethyl‐sulfinyl acetamide, has been proven as an effective agent against daytime fatigue caused by narcolepsy and obstructive sleep apnea (Battleday & Brem, 2016). The mechanism of action of modafinil is not yet completely understood; however, it is known to interact with systems of dopamine and norepinephrine, most likely resulting in the inhibition of reuptake of the respective catecholamines. As a result of the increase in overall cortical activity caused by this drug, a large sum of research has gone into trying to understand its potential for use as a cognitive enhancer and neuroprotective agent, as well as its properties as a stimulant (Hashemian & Farhadi, 2020). Several studies have reported the use of modafinil in the treatment of fatigue in MS, with some even analyzing its effects against other therapeutics such as l‐carnitine, amantadine, and cognitive behavioral therapy. The comparative safety and efficacy of these treatments, especially with consideration to cost‐friendliness, have been contested (Mücke et al., 2016).

Literature synthesizing data on the treatment of fatigue in MS with modafinil has been largely limited. A systematic review and meta‐analysis conducted by Shangyan et al. (2018) was able to consolidate much of the data that were available at the time and found positive effects of modafinil in decreasing fatigue. The objective of the present study is to assess the safety and efficacy of modafinil relative to l‐carnitine and placebo for the treatment of fatigue in patients with MS in light of more recent clinical trials. It serves to provide an update to the previous review with the consolidation of newer literature as well as the assessment of a variety of novel outcomes such as overall adverse events, QoL, Epworth Sleepiness Scale (**ESS)**, and subgroup comparisons, which have not previously been pooled in any other study as per our knowledge.

## 2 METHODS

### 2.1 Protocol and registration

The study protocol was registered via Prospero ([CRD42023491372], submitted on December 5, 2023).

### 2.2 Search strategy

This meta‐analysis was conducted according to the guidelines established by the Preferred Reporting Items for Systematic Review and Meta‐Analyses (PRISMA). An electronic search of PUBMED, ScienceDirect, and Cochrane Central was conducted for articles published from inception to December 2023 without any language restrictions using the following search string: “(Modafinil OR Provigil OR CRL‐40476 OR diphenylmethyl‐sulfinyl acetamide) AND (Fatigue OR Tiredness OR MFIS) AND (Multiple Sclerosis OR MS).’” Furthermore, we used pharmaceutical, generic, and trade names of modafinil to search for additional published and unpublished trials on clinicaltrials.gov. In addition, previously published meta‐analyses were also screened to identify any suitable studies matching the inclusion criteria.

### 2.3 Study selection

Studies were selected on the basis of the following inclusion criteria:

1. All studies must be controlled clinical trials or randomized controlled trials (RCTs)
2. Multiple‐sequence crossover studies must have an appropriate washout period of at least 2 weeks
3. All study subjects must be diagnosed with MS
4. The experimental group must be administered modafinil as an intervention
5. Studies must assess relevant quantitative outcomes of fatigue

Studies were excluded from the review on the basis of the following criteria:

1. Review articles and observational studies
2. Interventions using enantiomers or derivatives of modafinil (i.e., armodafinil)
3. Single‐arm studies and single‐sequence crossover studies
4. Studies where no relevant outcomes of fatigue are assessed
5. Studies ineligible for pooling (featuring comparator groups that exhibit distinct characteristics not evaluated in any other included study)
6. Irrelevant/Animal studies
7. Studies where data could not be retrieved

### 2.4 Data extraction and quality assessment

All studies identified by the searches and from additional sources were uploaded to EndNote to remove duplicates. All studies were screened by at least two reviewers (SUQ and SG). Disagreements were resolved via a third reviewer (MF). The remaining literature was compiled into an Excel spreadsheet and data were extracted, including the following study characteristics: name of study, year of publication, patient/population data, intervention information (dosage, duration, and measurement period), MS subtype, and outcome information. In studies employing scaled graphical representations as the primary means of data expression, quantitative outcomes were extrapolated through the application of photogrammetry. Additionally, efforts were made to obtain missing or raw data by contacting the study investigators when necessary. A risk of bias (RoB) assessment of studies was completed using the Cochrane RoB tool as well as the JBI critical appraisal checklist. The quality of evidence was assessed with the Grading of Recommendations, Assessment, Development, and Evaluation (GRADE) approach.

### 2.5 Outcomes

From the selected studies, the following outcomes of interest were extracted:

Primary outcomes:

1. Score on the Modified form of the Fatigue Impact Scale (MFIS)
2. Adverse events as defined by the total number of participants experiencing any of the following: vomiting, nausea, insomnia, injury, poisoning, nutritional, musculoskeletal, nervous, psychiatric, renal, respiratory, skin, subcutaneous, vascular disorders, or any event prompting a discontinuation of treatment
3. QoL, as measured through the following scales: Neuro‐QoL‐Fatigue Item Bank T‐score, MS QoL Inventory—General Health, Short Form 36 Physical Component Summary, and Hamburg QoL Questionnaire in MS

Secondary outcomes:

1. Score on the ESS
2. Fatigue Severity Scale (FSS)

### 2.6 Statistical analysis

All statistical analysis was performed on Review Manager (Version 5.4.1, Copenhagen: The Nordic Cochrane Centre, The Cochrane Collaboration, 2014). The outcomes were pooled using a random effects model. The random effects model assumes that different studies estimated different intervention effects, partly explaining the heterogeneity between studies. The statistical effects used were predicated upon the nature of the outcomes measured. For studies where a continuous outcome was measured using identical scales (i.e., change in baseline scores for MFIS), mean differences (MD) were extracted along with their 95% confidence interval. For continuous outcomes without standardized units (i.e., QoL), we extrapolated standardized mean difference (SMD) values. For dichotomous outcomes such as adverse events, risk ratios (RR) were used to pool data and evaluate effect size. In cases of zero events occurring in either the treatment or control groups when analyzing dichotomous outcomes, continuity corrections and appropriate weightage were applied to the studies to provide a stable estimate. We used the DerSimonian and Laird variance estimator for tau. Weightage of dichotomous variables such as mortality was assigned using the inverse variance method, The Higgins (*I* ^2^) statistic was used to evaluate heterogeneity, and a value of 25%–50% was considered low, 50%–75% as moderate, and >75% as high heterogeneity. The tolerated level of heterogeneity, meriting little further discussion, is set at less than or equal to 40%, a benchmark decided upon by reviewing the Cochrane Handbook. In all cases, a *p*‐value of .05 or less was considered significant. Publication bias was assessed via visual inspection of Begg's funnel plots.

## 3 RESULTS

### 3.1 Literature review

The PRISMA flow chart below summarizes the search and study selection process (Figure A). The initial search was conducted on December 1st on PubMed, ScienceDirect, and Cochrane Central, which yielded a total of 1260 results. After screening for relevance and removal of duplicates, 17 articles were assessed for eligibility. Among those, one was a letter to the editor, one was on the use of Armodafinil, one did not provide a relevant comparator group, and five studies were single‐arm, single‐sequence, or open‐label trials. A total of seven studies were included in the final analysis (Figure A).

**FIGURE A:**
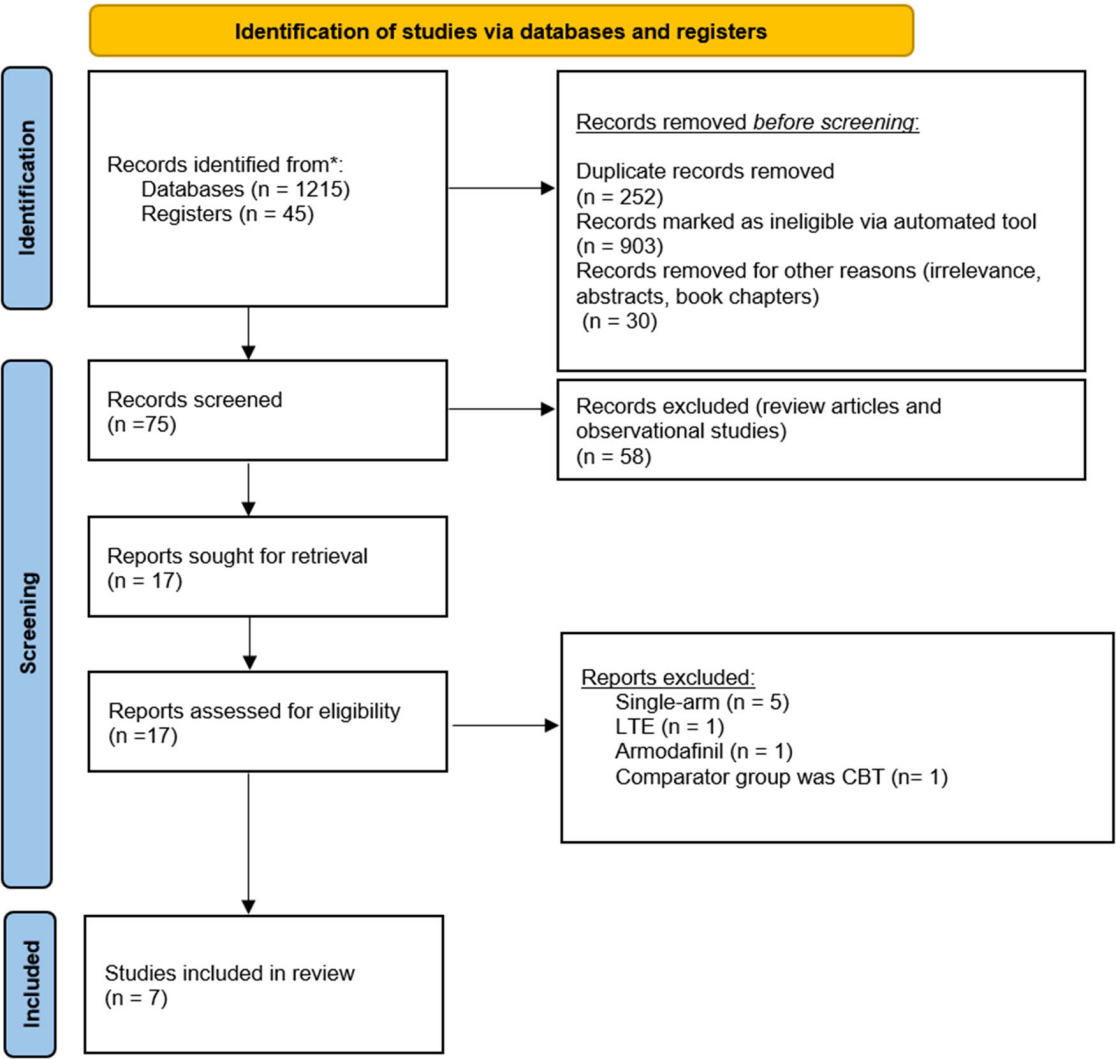
Preferred Reporting Items for Systematic Review and Meta‐Analyses (PRISMA) chart.

### 3.2 Study characteristics/baseline table

This study comprised a total of 486 participants. The study designs included in our analysis were predominantly RCTs (classic and crossover), along with a single unspecified clinical controlled trial. Amongst them, 297 were randomized to modafinil, whereas 293 were randomized to controls (35 to l‐carnitine and 263 to placebo); amounting to 590 unique data contributions across all study arms.^1^ The mean age of the included participants was 42.5 ± 9.2 years (Figure S1). We observed a predominantly female population (71%) (Figure S2). The dosages included for modafinil were primarily 200 mg with upward of 400 mg daily. The duration of treatment and measurement periods for the respective outcomes of the studies ranged from 2 to 8 weeks. The majority of patients had relapsing‐remitting MS (72%) (Figure S3). Baseline characteristics of the included studies are provided in Table S1.

### 3.3 Outcomes

#### 3.3.1 Primary

##### 3.3.1.1 MFIS

**FIGURE 1:**
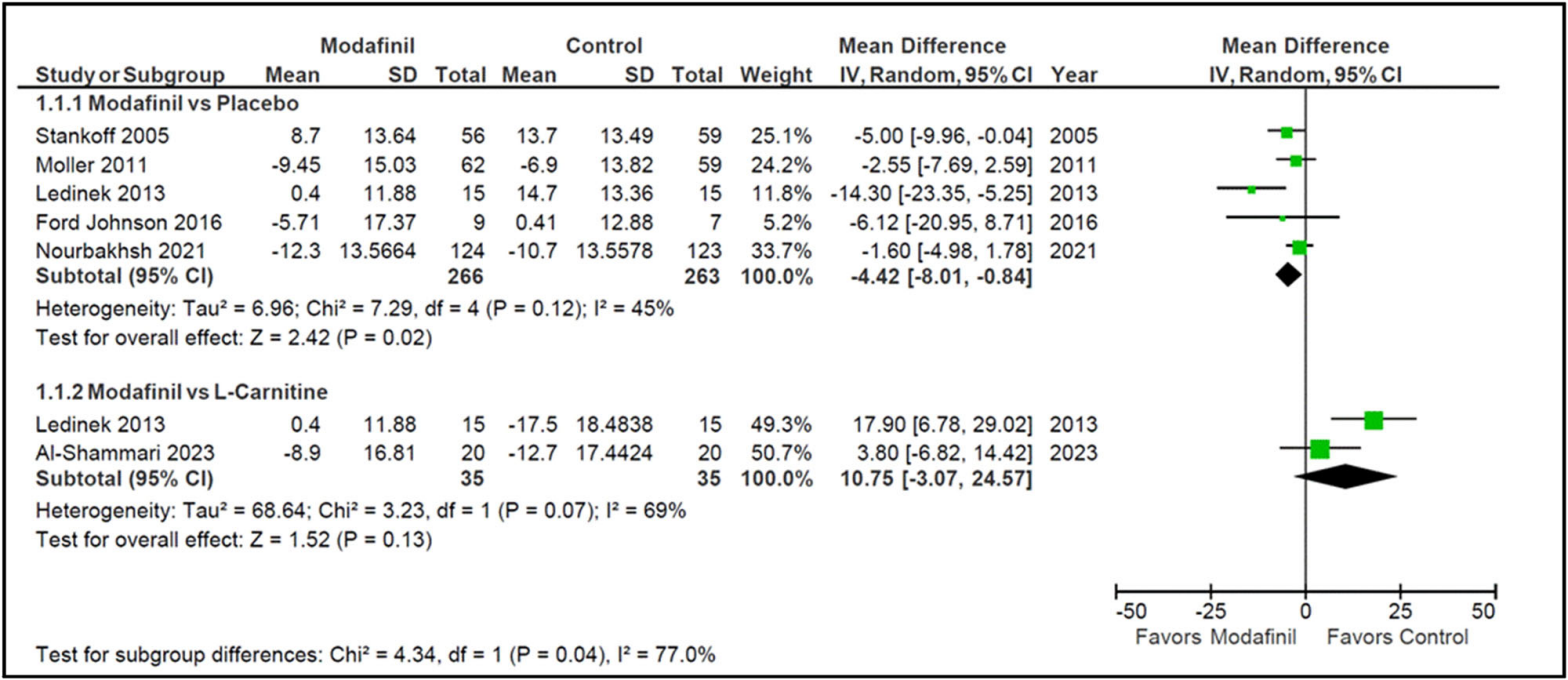
Modified form of the Fatigue Impact Scale (MFIS) (modafinil vs. placebo and modafinil vs. l‐carnitine).

Five studies assessed MFIS score difference from baseline between modafinil and placebo groups (Ford‐Johnson et al., 2016; Ledinek et al., 2013; Möller et al., 2011; Nourbakhsh et al., 2021; Stankoff et al., 2005). Treatment with modafinil led to a statistically significant reduction in MFIS scores compared to placebo (MD = −4.42 [−8.01, −.84]; *I* ^2 ^= 45%; *p* = .02). Only two studies compared modafinil treatment to l‐carnitine (Al‐Shammari et al., 2023; Ledinek et al., 2013). When compared with l‐carnitine, treatment with modafinil demonstrated higher MFIS scores on average, but the findings were not deemed to be statistically significant (MD = 10.75 [−3.07, 24.57]; *I* ^2 ^= 69%; *p* = .07). However, differences between the two subgroups were statistically significant (*p* = .04), implying that the difference in outcome between both comparators (l‐carnitine and placebo) was quite substantive. Heterogeneity was low among studies in the placebo subgroup (*I* ^2 ^= 45% and *p* = .12) and moderate among studies in the l‐carnitine subgroup (*I* ^2 ^= 69% and *p* = .07) (Figure 1).

##### 3.3.1.2 Adverse events

**FIGURE 2:**
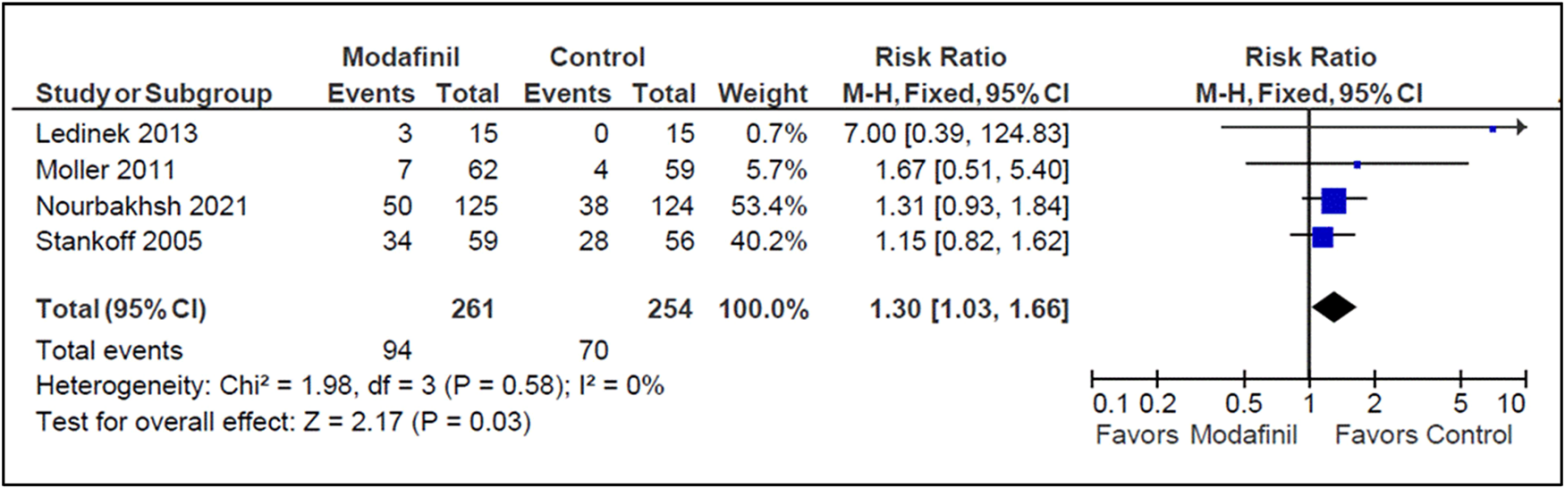
Adverse events (modafinil vs. placebo).

Four studies reported incidences of adverse events occurring during the treatment time (Ledinek et al., 2013; Möller et al., 2011; Nourbakhsh et al., 2021; Stankoff et al., 2005). Modafinil treatment had a higher risk of precipitating an adverse event compared to placebo (RR = 1.30 [1.03, 1.66]; *I* ^2 ^= 0%; *p* = .03). The most commonly reported adverse events were insomnia and gastrointestinal disturbances. Heterogeneity among studies was low (*I* ^2 ^= 0% and *p* = .58) (Figure 2).

##### 3.3.1.3 Quality of life

**FIGURE 3:**
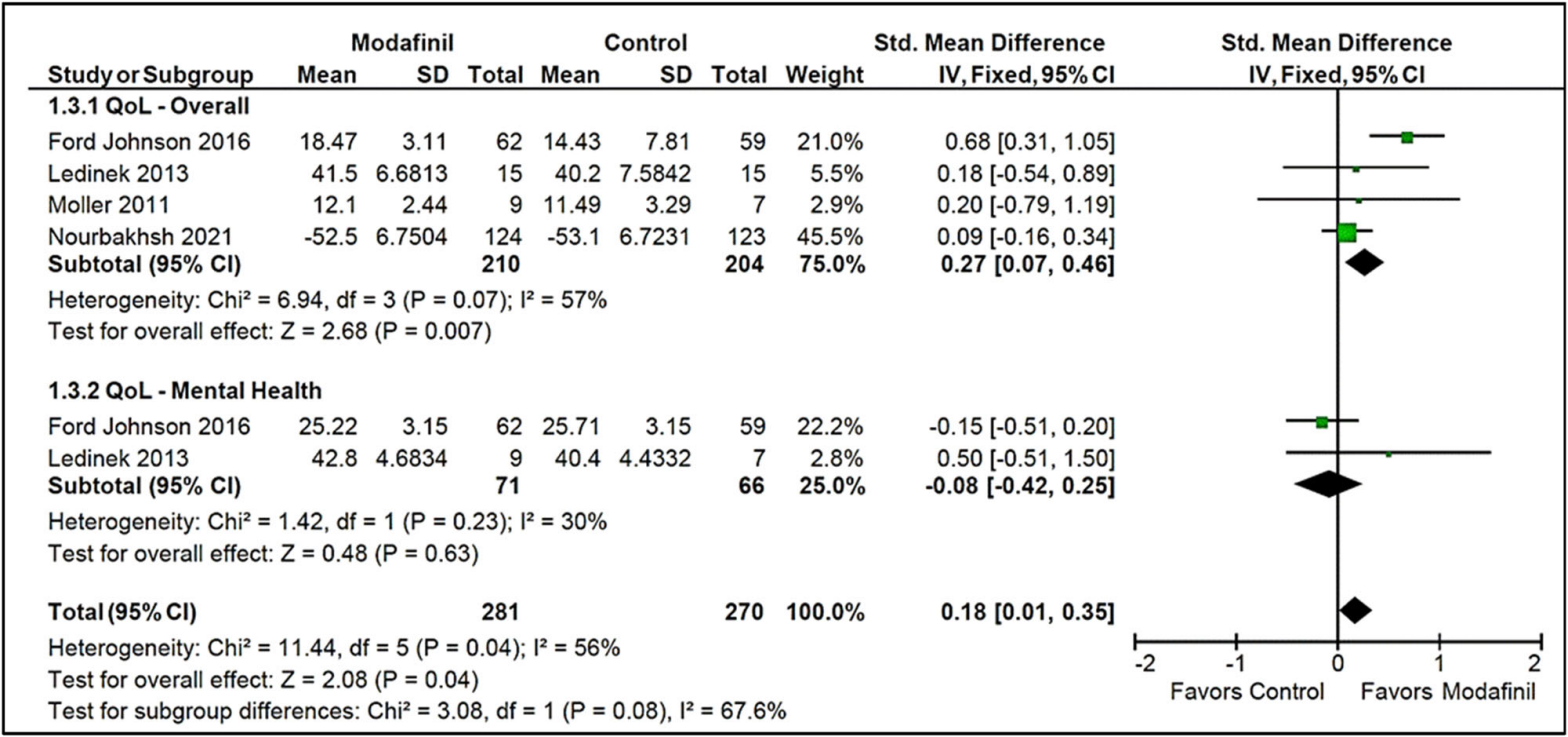
Quality of life (modafinil vs. placebo).

Four studies measured overall physical QoL scores posttreatment, and two studies measured mental‐health‐specific QoL sub‐scores (Ford‐Johnson et al., 2016; Ledinek et al., 2013; Möller et al., 2011; Nourbakhsh et al., 2021). Modafinil was found to contribute to an effectively higher overall physical QoL posttreatment than placebo (SMD = .27 [.07, .46]; *I* ^2 ^= 57%; *p* = .007). On the contrary, there was no significant difference observed in mental‐health‐specific aspects of QoL (SMD = −.08 [−.42, .25]; *I* ^2 ^= 30%; *p* = .63). After pooling the effects on both mental and physical health QoL measures, modafinil was found to have overall therapeutic benefit (SMD = .18 [.01, .35]; *I* ^2 ^= 56%; *p* = .04). A statistically significant difference between the subgroups was not found (*p* = .08). Study heterogeneity was moderate in the “Overall [Physical]” subgroup (*I* ^2 ^= 57%, *p* = .07) and low in the “Mental Health” subgroup (*I* ^2 ^= 30% and *p* = .23) (Figure 3).

#### 3.3.2 Secondary

##### 3.3.2.1 ESS

**FIGURE 4:**
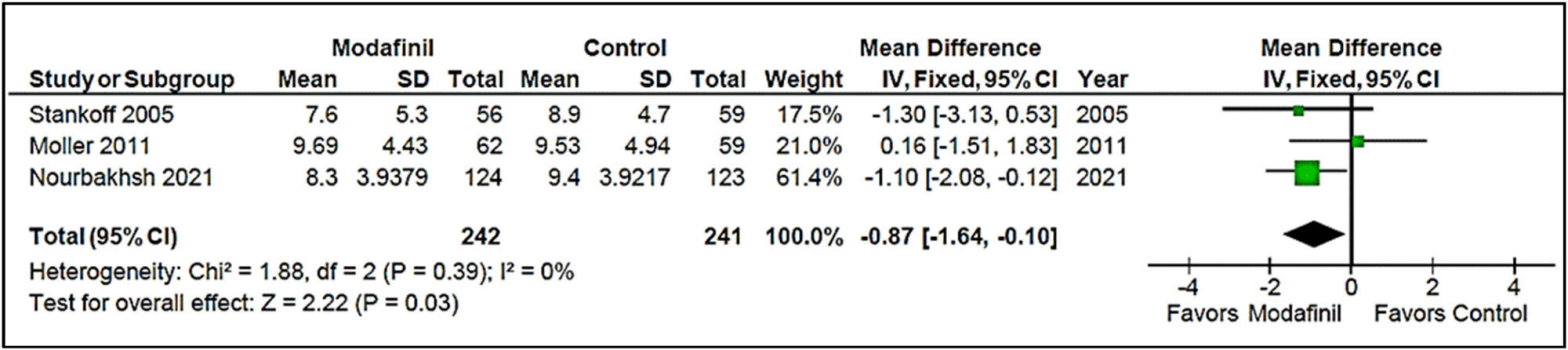
Epworth Sleepiness Scale (ESS) (modafinil vs. placebo).

Three studies measured change from baseline scores on the ESS (Möller et al., 2011; Nourbakhsh et al., 2021; Stankoff et al., 2005). Modafinil contributed to a minor but statistically meaningful reduction in ESS scores (MD = −.87 [−1.64, −.10]; *I* ^2 ^= 0%; *p* = .03). Heterogeneity among studies was low (*I* ^2 ^= 0% and *p* = .39) (Figure 4).

##### 3.3.2.2 FSS

**FIGURE 5:**
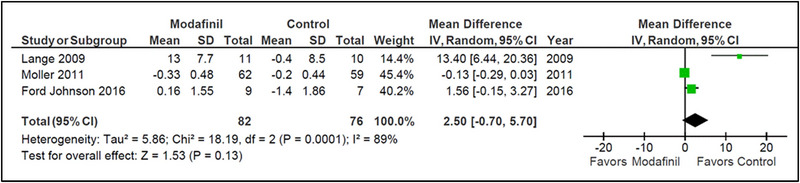
Fatigue Severity Scale (FSS) (modafinil vs. placebo).

Three studies measured change from baseline scores or final scores on the FSS (Ford‐Johnson et al., 2016; Lange et al., 2009; Möller et al., 2011). No statistically significant difference was found between the modafinil and placebo groups (MD = 2.5 [−.70, 5.70]; *I* ^2 ^= 89%; *p* = .13). Heterogeneity among studies was high (*I* ^2 ^= 89% and *p* = .0001). However, the greatest contribution of heterogeneity came from Lange et al., the removal of which would still result in statistically insignificant findings with only moderate heterogeneity (*I* ^2 ^= 73% and *p* = .05) (Figure 5).

### 3.4 Quality assessment and publication bias

**FIGURE 6:**
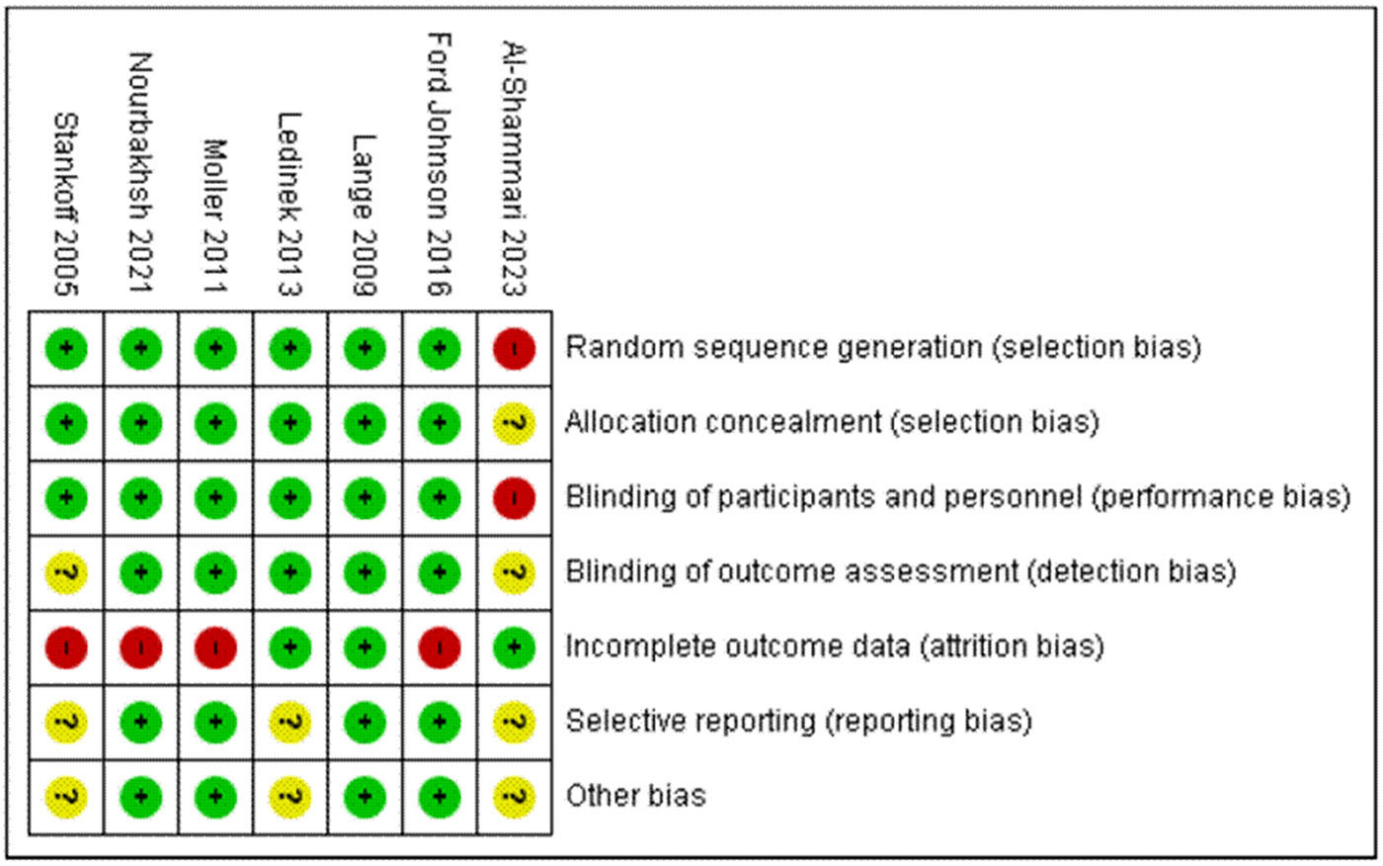
Risk of bias (RoB) summary plot.

**FIGURE 7:**
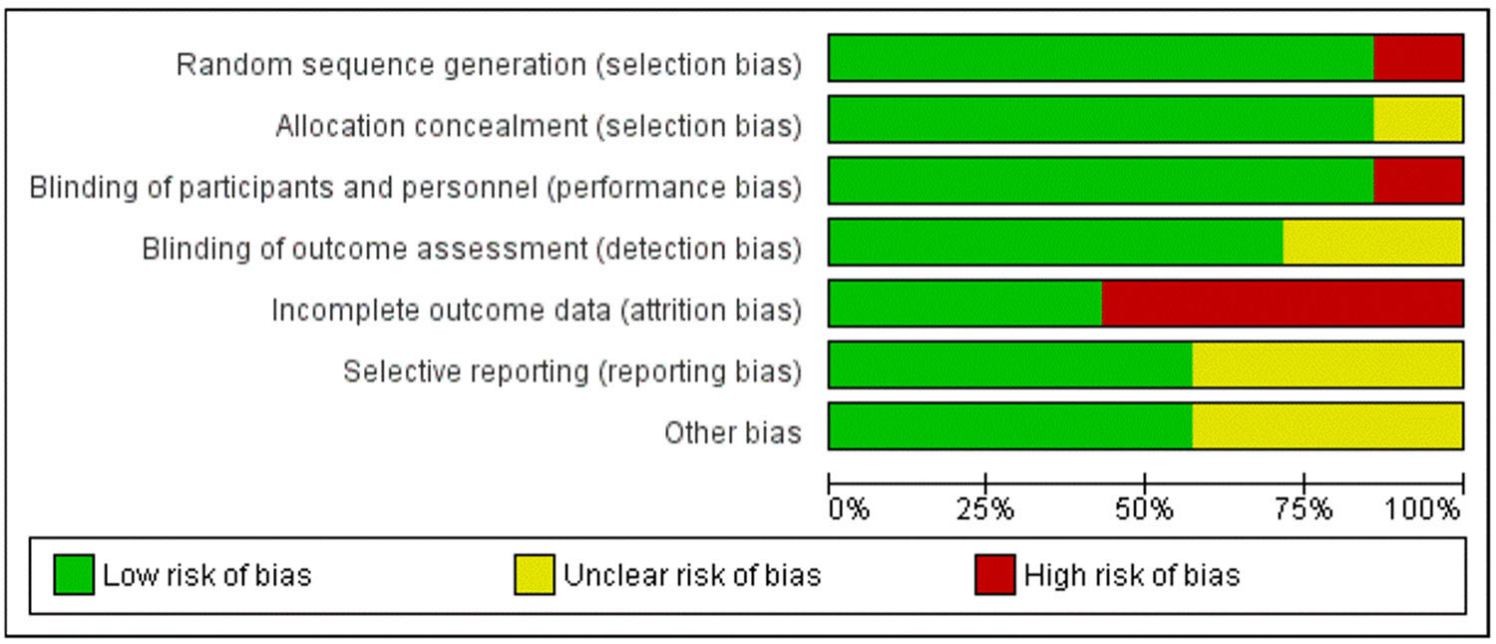
Risk of bias (RoB) summary chart.

**FIGURE 8:**
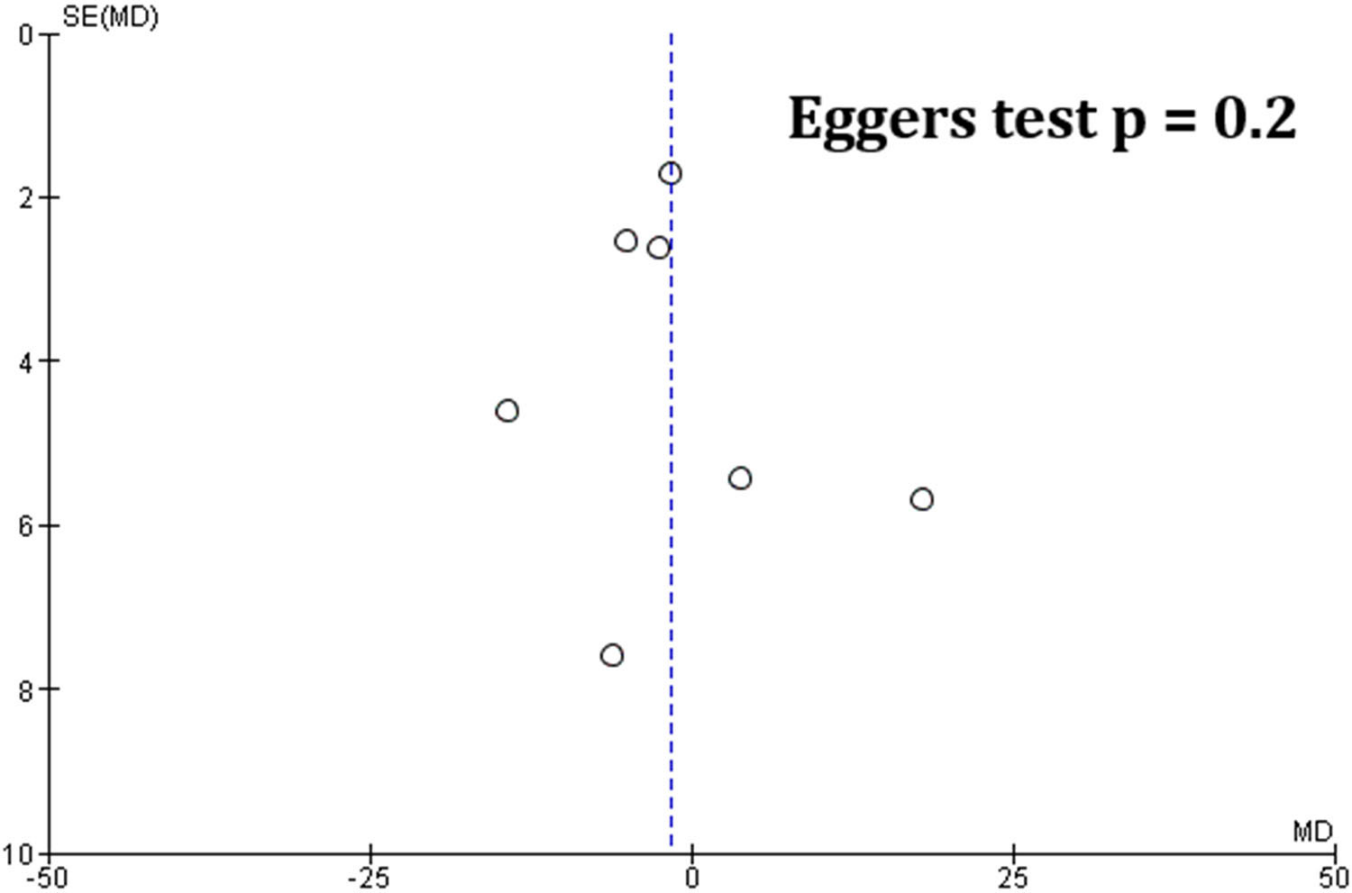
Eggers plot for publication bias.

The quality of studies was assessed using the Cochrane RoB tool as well as the 2020 JBI Critical Appraisal Checklist. The overall quality of evidence of the included studies was judged to be moderate. The included studies were at the highest risk of attrition bias due to patient dropout/discontinuation, which had occurred in four studies (Ford‐Johnson et al., 2016; Möller et al., 2011; Nourbakhsh et al., 2021; Stankoff et al., 2005). Only Al‐Shammari et al. (2023) did not report random sequence generation, allocation concealment, or blinding of participants. Blinding of outcome assessment was unclear in Al‐Shammari et al. (2023) and Stankoff et al. (2005). Selective reporting and other biases were unclear in Stankoff et al. (2023), Ledinik et al. (2013), and Al‐Shammari et al. (2005) due to limited raw data reported in the findings. RoB graph and summary of findings plot are provided in Figures 6 and 7. A table summary of the JBI critical appraisal checklist is provided in Table S2. Egger's regression test for funnel plot asymmetry was nonsignificant indicating no significant risk of publication bias (Figure 8). A summary of findings table using the GRADE approach is provided as Table S3.

## 4 DISCUSSION

Our findings, across seven studies, demonstrate that modafinil leads to a statistically meaningful reduction in fatigue when compared with placebo, as measured by MFIS and ESS. These findings coincide with previous meta‐analyses, such as Shangyan et al. (2018), which included only four studies for the assessment of MFIS. In addition, modafinil demonstrated a greater risk of precipitating adverse events as well when compared with placebo, which differs from the findings of Yang et al. (2017) who analyzed the effects of modafinil on study discontinuation due to adverse events and discovered no meaningful differences between the groups. l‐Carnitine, which was used as a comparator in two studies, was generally found to be more efficacious than modafinil in reducing MFIS scores, though the findings were not statistically significant. Overall, the most common drugs prescribed for the treatment of fatigue in MS include amantadine, modafinil, methylphenidate, and l‐carnitine. Although multiple studies have compared modafinil with amantadine and methylphenidate, there have been no previous meta‐analyses comparing l‐carnitine with modafinil for fatigue in MS. Prior studies comparing l‐carnitine with amantadine demonstrated that they both hold similar efficacy. However, these findings were pooled using SMDs due to heterogeneous metrics of fatigue, and the most recent crossover study included in our analysis found superior effects with amantadine, followed by l‐carnitine, methylphenidate, and modafinil, respectively (Nourbakhsh et al., 2021).

Fatigue in MS is a complex, multidimensional condition that drastically affects the social and professional aspects of patients’ lives. According to the MS Council of Clinical Practice Guidelines, it is officially described as “a subjective lack of physical and/or mental energy that is perceived by the individual or caregiver to interfere with usual and desired activities.” There are three distinct cognitive domains in fatigue: the physical, the cognitive, and the psychosocial (Ayache et al., 2022). To our knowledge, this study is the first to shed light on the distinct effects of modafinil in MS patients within these domains via a pooled subgroup analysis of QoL outcomes. Our study found contrasting effects of modafinil on physical and mental/psychosocial QoL indicators. One potential reason for this finding may be that, although modafinil improves physical function by promoting wakefulness, the resulting adverse effects such as insomnia may contribute to a deterioration of mental and psychological well‐being over time (Wisor, 2013). The effects of various treatment modalities on different domains of fatigue might serve as an important avenue for future research.

Despite our best efforts to ensure the generalizability and reproducibility of our findings, there remain several limitations to our study. One such limitation is the presence of publication bias due to non‐publication of studies that did not demonstrate statistically significant findings, which was established via a visual inspection of Briggs funnel plot. In addition, clinical heterogeneity among the study designs persists, with differences in dosage and treatment duration throughout the trials. This may be compounded by the fact that certain outcomes, such as QoL, were measured using various scales; although a conversion to SMD as an effect measure helped standardize these metrics, the clinical interpretation of these findings may be less straightforward. Furthermore, several studies conferred a high risk of attrition bias due to patient dropout, and one study did not specify any protocol for randomization or blinding of participants. Ultimately, more precise RCTs with higher statistical power and greater sample sizes are required to provide superior insight and improved quality of evidence for this intervention.

## 5 CONCLUSION

Ultimately, our meta‐analysis expands upon the previous literature by (1) including studies published until December 2023, (2) pooling outcomes such as ESS, adverse events, and QoL measures, and (3) by comparing modafinil with l‐carnitine. Thus, we believe our study serves as the most comprehensive quantitative review of the safety and efficacy of modafinil for the treatment of fatigue in patients with MS to date. The data indicate that modafinil confers a therapeutic benefit in treating fatigue in patients with MS and improves overall QoL; however, there is a heightened risk of precipitating adverse events such as insomnia and gastrointestinal symptoms. Future research comparing modafinil to other treatment modalities as well as pooling of more precise studies with similar intervention characteristics would be required to better inform clinical management.

## AUTHOR CONTRIBUTIONS

**Shamas Ghazanfar**: Conceptualization; data curation; formal analysis; investigation; methodology; resources; software; supervision; validation; visualization; writing—original draft; writing—review and editing. **Minaam Farooq**: Conceptualization; investigation; resources; writing—original draft. **Shurjeel Uddin Qazi**: Data curation; formal analysis; methodology; software; validation; writing—review and editing. **Bipin Chaurasia**: Investigation; resources; writing—review and editing. **Ulrike Kaunzner**: Conceptualization; supervision; validation; writing—original draft; writing—review and editing.

## FUNDING INFORMATION

No funds, grants, or other support were received.

## CONFLICT OF INTEREST STATEMENT

On behalf of all authors, the corresponding author states that there is no conflicts of interest.

### PEER REVIEW

The peer review history for this article is available at https://publons.com/publon/10.1002/brb3.3623.

## Supporting information

Supporting Information

Table S1

Table S2: JBI critical assessment of included studies

Table S3: GRADE summary of findings

## Data Availability

The data that support the findings of this study are openly available in the included studies.

## NOTE

1 This was due to the presence of crossover RCTs, in which participants contributed to both the control and intervention groups after an appropriate 2‐week washout period.
