# Supplementary material for: The use of modafinil for the treatment of fatigue in multiple sclerosis: A systematic review and meta‐analysis of controlled clinical trials
Source: Brain Behav. 2024 Jul 10;14(7):e3623. doi: 10.1002/brb3.3623 (PMC11237168; doi:10.1002/brb3.3623)
Supplement: Supplementary file 1 — Supporting Information [file BRB3-14-e3623-s004.docx]

**Supplementary Figure 1**

**Supplementary Figure 2**

**Supplementary Figure 3**
