# Supplementary material for: The use of modafinil for the treatment of fatigue in multiple sclerosis: A systematic review and meta‐analysis of controlled clinical trials
Source: Brain Behav. 2024 Jul 10;14(7):e3623. doi: 10.1002/brb3.3623 (PMC11237168; doi:10.1002/brb3.3623)
Supplement: Supplementary file 4 — Table S3: GRADE summary of findings [file BRB3-14-e3623-s002.docx]

| **Supplementary Table 1:** Certainty of evidence assessment using the GRADE (Grading of Recommendations, Assessment, Development and Evaluation) approach. | | | | | | | | | | |
| --- | --- | --- | --- | --- | --- | --- | --- | --- | --- | --- |
| **Outcome** | **Number of studies** | **Study design** | **Risk of Bias** | **Inconsistency** | **Indirectness** | **Imprecision** | **Publication Bias** | **Other** | **Effect Size** | **Certainty** |
| **MFIS** | 6 | RCTs | Not serious | Not serious | Not serious | Not serious | Undetected | None | -1.70 [-6.74, 3.34] | ⨁⨁⨁⨁  High |
| **Adverse events** | 4 | RCTs | Not serious | Serious | Not serious | Serious | Undetected | None | 1.30 [1.03, 1.66] | ⨁⨁⨁ Moderate |
| **Quality of Life** | 4 | RCTs | Not serious | Serious | Serious | Not serious | Undetected | None | 0.18 [0.01, 0.35] | ⨁⨁⨁ Moderate |
| **ESS** | 3 | RCTs | Not serious | Serious | Serious | Serious | Undetected | None | -0.89 [-1.66, -0.12] | ⨁⨁  Low |
| **FSS** | 3 | RCTS | Not serious | Not serious | Serious | Serious | Undetected | None | 2.50 [-0.70, 5.70] | ⨁⨁  Low |
| RCTs, Randomized Controlled Trials, CI, Confidence Intervals. The GRADE approach is a methodical and transparent system used to assess the certainty level of evidence in systematic reviews. It uses eight criteria to assess the quality of outcomes such as the design of studies included in the outcomes, risk of bias of studies, inconsistencies in reporting the results by the studies, how well the studies answer the review question, how precise is the effect size and the publication bias of the outcome. | | | | | | | | | | |
